# Mindful Attention and Pain Appraisal During Isometric Exercise

**DOI:** 10.3390/bs16050709

**Published:** 2026-05-05

**Authors:** Sara A. Thompson, Sarah Ullrich-French, Anne E. Cox

**Affiliations:** 1 Faculty of Kinesiology and Physical Activity, University of Toronto, Toronto, ON M5S 1A1, Canada; 2 College of Education, Sport, and Human Sciences, Washington State University, Pullman, WA 99164, USA; anne.cox@wsu.edu

**Keywords:** mindfulness, attentional focus, pain perception, affect

## Abstract

Exercise-induced pain is a common and aversive experience that can influence how individuals engage with and persist in physical activity. Pain is not solely determined by sensory input but is shaped by attentional and cognitive processes that influence how bodily sensations are interpreted during exercise. The present study examined how different modes of attentional engagement shape the cognitive appraisal of exercise-induced pain. Recreationally active adults (N = 55) were randomly assigned to use either a mindful associative attentional strategy or a cognitively engaging dissociative strategy (backward counting) during two isometric endurance tasks (forearm plank and wall-sit). Core affect, pain severity, pain tolerance, and mindful reappraisal of pain were assessed during and after exercise. Manipulation checks confirmed robust differentiation of attentional focus and state mindfulness between conditions (*p* < 0.05). Participants using mindful attention reported significantly higher mindful reappraisal of pain during both exercises (*p* < 0.05, η^2^ = 0.11 (plank) and 0.11 (wall sit)) and lower pain severity during the plank only (*p* < 0.05, η^2^ = 0.08 (plank)) compared to those using dissociative attention. No between-condition differences were observed for pain tolerance, perceived exertion, or core affect. These findings suggest that mindful attention may influence exercise-induced pain in part through differences in cognitive appraisal rather than disengagement from bodily sensations. By highlighting pain appraisal as a key attentional mechanism, this study contributes to a more precise understanding of how mindfulness shapes pain experiences during acute exercise.

## 1. Introduction

Sustaining regular engagement in physical activity remains a persistent challenge for many individuals, with pain and discomfort among the most frequently cited barriers to continued participation. During moderate- to high-intensity exercise, individuals commonly experience exercise-induced pain (EIP), a transient and non-pathological sensation arising from nociceptive signaling associated with metabolic byproducts, mechanical pressure, and sustained muscle contraction (O’Connor & Cook, 2001; Pollak et al., 2014). Although EIP does not reflect tissue damage, it is often interpreted as aversive and can influence affective responses, motivation, and decisions to persist or disengage from exercise (Rhodes & Kates, 2015; Rice et al., 2019). Understanding how individuals process and interpret pain during exercise is therefore critical for clarifying the psychological mechanisms that shape exercise experiences and behavior.

Pain is not purely a sensory phenomenon but a dynamic perceptual experience that interacts with attentional processes. Experimental and clinical models consistently demonstrate that pain captures attention, alters information processing, and biases cognitive resources toward bodily threat signals (McMorris et al., 2018; Stevens et al., 2018). In exercise contexts, this attentional competition is especially relevant as sustained physical effort requires ongoing monitoring of bodily signals, yet excessive attention to discomfort may amplify pain perception and undermine task engagement. These findings suggest that during exercise, the way attention is engaged may be as important as where attention is directed in shaping how pain is experienced.

Contemporary accounts of perception further emphasize that pain is not passively received but actively constructed through ongoing interpretive processes. Frameworks such as predictive processing propose that the brain continuously generates expectations about bodily states and updates these expectations based on incoming sensory input (Friston, 2010). Within this view, pain reflects an interaction between nociceptive signals and prior expectations about threat and bodily integrity. Attentional engagement may therefore influence pain not only by allocating processing resources, but by shaping how sensory input is interpreted and weighed. This perspective provides a broader theoretical context for understanding how different modes of attention may alter the experience of EIP.

Attentional strategies used during exercise are often characterized along an associative–dissociative continuum. Dissociative strategies, such as distraction or external focus, direct attention away from bodily sensations, whereas associative strategies involve attending to internal cues such as breathing, muscle tension, or effort (Lind et al., 2009; Brick et al., 2014). Distraction is frequently effective for reducing discomfort in untrained individuals, particularly when exercise sensations are unfamiliar or overwhelming (Brick et al., 2014). However, for individuals with regular exercise experience, internally focused strategies may support task engagement, performance monitoring, and persistence by facilitating adaptive interpretation of interoceptive signals (di Fronso et al., 2018; Birrer & Morgan, 2010). Importantly, these effects are not limited to elite or professional athletes but extend to recreationally active adults who meet recommended physical activity guidelines. This raises the possibility that mindfulness influences pain during exercise not by disengaging attention away from bodily sensations, but by changing how those sensations are cognitively interpreted.

Mindfulness represents a distinct form of associative attention characterized by present-moment awareness and non-reactivity to internal experiences (Kabat-Zinn, 1990), which can support emotional regulation, and exercise performance (Davis & Hayes, 2011; Chen et al., 2018). Unlike attentional monitoring that emphasizes evaluation or control, mindful attention encourages observation of bodily sensations without judgment or avoidance. Theoretically, this mode of attention may alter experiences not by disengaging from discomfort, but by changing how pain-related sensations are cognitively appraised, allowing sensations to be experienced without being amplified by negative evaluation or threat-based interpretation. According to the Mindfulness-to-Meaning Theory, mindfulness facilitates a shift away from automatic negative appraisals toward more flexible, non-threatening interpretations of discomfort, thereby interrupting maladaptive cognitive affective cycles that amplify pain (Garland et al., 2015, 2022). Evidence from experimental pain paradigms support this account, showing mindfulness-based strategies can reduce pain intensity and increase pain tolerance, even following brief interventions (Kingston et al., 2007; Wang et al., 2019). In exercise settings, acute mindfulness manipulations have been shown to support positive affective responses comparable to distraction-based strategies (Cox et al., 2020). However, much of the existing literature emphasizes observable outcomes such as affective responses or pain tolerance, while providing limited insight into the cognitive mechanisms through which mindfulness shapes pain experiences during exercise. In particular, it remains unclear whether mindfulness influences exercise-induced pain by disengaging attention from discomfort, or by altering the cognitive appraisal of pain while maintaining engagement with bodily sensations.

The Mindful Reappraisal of Pain Scale (MRPS) was developed to quantify individual differences in the cognitive reappraisal of pain, rather than indexing general mindfulness or pain coping broadly. The MRPS captures the extent to which individuals reinterpret pain sensations in a non-judgmental, non-threatening manner, reflecting the cognitive reappraisal processes theorized to underline mindfulness-based analgesia (Garland et al., 2022). Applying the MRPS within an exercise context provides a novel opportunity to examine whether mindful attention specifically alters pain appraisal during physically demanding tasks, rather than merely changing affective valance or performance outcomes.

The present study examined how different modes of attentional engagement shape the cognitive appraisal of exercise-induced pain. Specifically, we compared a mindful associative strategy with a cognitively engaging dissociative strategy involving backward counting during two isometric endurance tasks in recreationally active adults.

Isometric tasks were selected because they reliably elicit exercise-induced pain while minimizing extraneous performance demands, allowing for clearer examination of attentional mechanisms. By comparing mindfulness with an active, cognitively engaging distraction strategy, this study aimed to isolate differences in how attention is deployed, rather than whether attention is occupied. The primary outcome of interest was state mindful reappraisal of pain, assessed using the MRPS, to evaluate whether mindful attention was associated with differences in pain appraisal during exercise. Secondary outcomes include pain severity, pain tolerance, and core affect, providing contextual information about how changes in appraisal relate to experiential and behavioral responses. We hypothesized that mindful attention would be associated with greater mindful reappraisal of pain compared to distraction. Given prior findings that mindfulness and distraction can support similar affective experiences during exercise (Cox et al., 2020; Kingston et al., 2007), we expected affective responses to be comparable across conditions and that differences in pain tolerance would be minimal. By isolating how attention is engaged during exercise, this study aims to clarify how attentional strategies relate to the cognitive appraisal of exercise-induced pain.

## 2. Method

### 2.1. Participants

Fifty-seven participants began the study, with two participants withdrawing due to scheduling conflicts, resulting in a final sample of 55 participants aged 18 to 43 years (*M* = 22.05, *SD* = 5.54). Participants self-identified as female (54%), White (61.8%), Asian/Pacific Islander (18.2%), Multiracial (10.9%), Hispanic/Latino (7.3%), and Other (1.8%). Participants were randomly assigned to either a mindfulness (associative) attentional strategy (*n* = 28) or a backward counting (dissociative) strategy (*n* = 27). Inclusion criteria included being between 18 and 65 years of age, not being pregnant, meeting current physical activity guidelines (at least 150 min of moderate-intensity activity or 75 min of vigorous-intensity activity a week for at least 6 months), and having no contraindications to physical activity. Physical activity levels were assessed using the International Physical Activity Questionnaire—Short form (Craig et al., 2003), and readiness for physical activity was assessed using the Physical Activity Readiness Questionnaire (Warburton et al., 2011). Demographic characteristics and baseline variables (see Table 1) did not significantly differ by condition (*p* > 0.05). This study was conducted in accordance with the Declaration of Helsinki and approved by the Institutional Review Board of Washington State University (#200112-001 approved 15 September 2023). Participants were recruited through flyers, online postings, and in-person recruitment. Participants provided informed consent prior to participation and were compensated $10 for each session completed.

A priori power analysis was conducted using moderate to high effect sizes (g = 0.68) reported for mindfulness-based effects on pain tolerance in acute pain contexts (Shires et al., 2020) and high effect size (Cohen’s D = 1.51) reported on the sensitivity of the Mindful Reappraisal of Pain Scale to mindfulness (Garland et al., 2022). A G*Power (version 3.1.9.7) calculation for an independent samples *t*-test (α = 0.05, power = 80, and a moderate-to-large effect size *d* = 0.68) indicated a required sample size of 56 participants for *t*-tests, and calculation for ANCOVA (α = 0.05, power = 80, and a large effect size *f* = 0.50) indicated a required sample size of 42, supporting the adequacy of the final sample size.

### 2.2. Baseline Measures

Trait Mindfulness. The 15-item short-form Five Facet Mindfulness Questionnaire (FFMQ-SF) assessed participants’ mindfulness propensity in daily life (Baer et al., 2006). Items were rated on a 5-point Likert scale and averaged to produce a composite mindfulness score. The FFMQ-SF has demonstrated adequate reliability and validity in prior research (Gu et al., 2016), which showed acceptable internal consistency in the present sample (α = 0.75).

Daily Pain. Perceived daily pain and pain-related interference over the previous four weeks were assessed using two items from the Short-Form 36 Bodily Pain Scale (SF-36 BPS) (Ware & Sherbourne, 1992). The SF-36 BPS is widely used and has demonstrated strong psychometric properties (Hawker et al., 2011).

Trait Mindful Reappraisal of Pain. The Mindful Reappraisal of Pain Scale (MRPS: Garland et al., 2022), adapted from the Coping Strategies Questionnaire, measures the tendency to reinterpret pain sensations in a non-judgmental and adaptive manner. The MRPS has demonstrated evidence of convergent validity and reliability (Garland et al., 2022) and showed good internal consistency in this study (α = 0.81).

### 2.3. Outcome Measures

Affective Responses. Participants rated pleasure or displeasure during exercise using the Feeling Scale (FS: Hardy & Rejeski, 1989), ranging from +5 (very good) to −5 (very bad). Affect was assessed before, during, and after each exercise bout. Scores assessed every 20 s during each exercise were averaged to represent one score during exercise. The FS has demonstrated validity for assessing affective responses during physical activity (Tenenbaum et al., 2007).

Pain Severity. Pain intensity during exercise was assessed using the Numeric Rating Scale (NRS; Williamson & Hoggart, 2005), an 11-point scale ranging from 0 (no pain at all) to 10 (worst pain imaginable). Scores during each exercise were averaged. The NRS is well-validated and commonly used in exercise and experimental pain contexts (Hawker et al., 2011).

Exercise Pain Tolerance. Pain tolerance was operationalized as the maximum hold time (in seconds) for each isometric exercise (forearm plank and wall-sit). Trials were terminated when participants voluntarily stopped or failed to maintain proper form, as determined by trained research assistants. Maximum hold time is commonly used as an index of pain tolerance and muscular endurance in isometric tasks (Frey Law et al., 2010; El ahrache et al., 2006). To account for individual differences in baseline tolerance, changes in maximum hold time from baseline to experimental session were also analyzed.

State Mindful Reappraisal of Pain. State mindful pain reappraisal was assessed using a modified version of the MRPS adapted to capture in-task experiences (Garland et al., 2022; Thompson et al., 2025). Instructions were adapted to state “please select the response that indicates how frequently you implemented each statement during the exercise you just completed.” Items were modified to reflect past tense instead of present tense to further direct them to assess mindful reappraisal of pain in response to the exercise they just completed (e.g., “I watch my pain from a distance” was changed to “I watched my pain from a distance”). The scale contains nine items rated on a 7-point scale ranging from 0 (never) to 6 (always). Higher scores reflect great mindful reappraisal of pain. Participants completed the same items immediately after completing each exercise resulting in two independent scores. Internal consistency reliability was good across exercises (α = 0.87–0.91). Preliminary validity evidence for the state-adapted MRPS is promising (Thompson et al., 2025).

Perceived Exertion. The Borg Ratings of Perceived Exertion (RPE) Scale was used to assess perceived exertion (Borg, 1982). Participants rated how hard they felt they were working immediately following each exercise. The RPE scale has demonstrated strong validity for assessing perceived effort in adults (Borg, 1985).

### 2.4. Manipulation Check Variables

State Mindfulness. The State Mindfulness Scale for Physical Activity 2 (SMSPA-2) was used to measure how mindful participants were of their physical and mental experience during each respective isometric exercise using a 5-point scale (Ullrich-French et al., 2021). Total scores reflected participants’ momentary monitoring and acceptance of physical and mental experiences. The SMSPA-2 has demonstrated strong psychometric properties (Ullrich-French et al., 2021), and the internal consistency reliability in this study was good (α = 0.80–0.93).

Attentional Focus. The Attentional Focus Scale was used to assess the individual’s attentional focus during the duration of the exercise bout using a 10 cm line with one end labeled internal focus (heart rate, breathing, bodily sensations) and the other end labeled external focus (daydreaming, external environment) (Tammen, 1996). Lower scores indicated greater internal focus, whereas higher score reflected greater external focus. Tammen (1996) provides evidence supporting the use of this scale.

### 2.5. Procedure

This study employed a randomized between-subjects experimental design with two laboratory-based sessions separated by 48 h (2 days). The first session served as a baseline assessment and included eligibility screening, informed consent, instruction of exercise form, and completion of baseline measures. Baseline exercise performance was used as a within-person reference point to characterize participants’ typical attentional engagement in the absence of explicit strategy instruction.

Participants completed two isometric endurance tasks—a forearm plank and a wall-sit, selected to elicit reliable exercise-induced pain while minimizing extraneous skill demands. Exercise order was randomized and remained consistent across sessions. Standardized verbal and visual instructions were provided prior to each task, and participants completed a brief five-second practice to ensure proper form without inducing fatigue. Participants were instructed to maintain each exercise position as long as possible while preserving proper form. Trials were terminated when participants voluntarily stopped or failed to correct improper exercise form within five seconds following a verbal cue. Each exercise was performed once and a minimum four-minute rest period was provided between exercises, and participants rested until heart rate returned to baseline (Denis et al., 2022).

**Figure 1:**
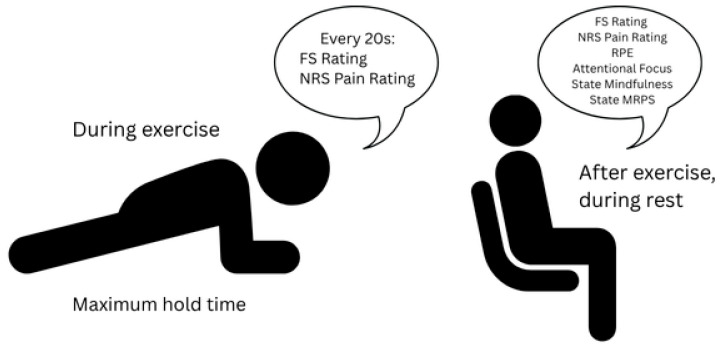
Procedure and timing of measurements during the forearm plank task (identical procedure used for the wall-sit). FS (Feeling Scale) and NRS (pain severity) were assessed every 20 s during the task and then averaged for analysis. RPE (Rating of Perceived Exertion), post-task affect, and MRPS (Mindful Reappraisal of Pain Scale) were assessed immediately following each task completion and analyzed as post-task values. Rest time was a minimum of 4 min.

During each exercise, affect (FS) and pain severity (NRS) were assessed every 20 s. Immediately following each exercise, participants completed Ratings of Perceived Exertion (RPE), post-exercise affect, pain severity, and state mindful reappraisal. See Figure 1 for the in-task measurement procedure. At the conclusion of the first session, participants were informed of their assigned attentional strategy and instructed on how to practice it prior to the second session. Participants were asked to refrain from practicing the plank or wall-sit between sessions. For analyses, pain severity and affect were summarized as mean values across the duration of each exercise bout, while post-task ratings (RPE and state mindful reappraisal) were analyzed as single scores following each task.

#### 2.5.1. Mindfulness Condition

Participants assigned to the mindfulness condition received standardized instruction emphasizing present-moment awareness and non-judgmental attention to bodily sensations. A brief guided grounding mindfulness exercise was completed at the end of the first session, and participants were provided with a guided body scan audio to practice between sessions to support familiarity with mindful attention.

During the experimental session, participants were reminded to adopt a mindful attentional focus and were provided with brief verbal cues every 10 s (e.g., “Notice the sensations in your core”, “allow the breath to move naturally”). These cues were designed to support sustained mindful engagement without directing participants to suppress or avoid discomfort.

#### 2.5.2. Backward-Counting Condition

Participants assigned to the backward counting condition were instructed to count backward aloud by sevens during each exercise bout. This strategy was selected to provide an active, cognitively engaging form of distraction. Backward counting is in line with other cognitive dissociative strategies used during exercise (Gould et al., 1980; Paul et al., 2021). Participants practiced backward counting between sessions and received daily text prompts with random starting numbers as practice.

During the experimental session, participants were cued with a new starting number every 10 s and instructed not to prioritize accuracy, but to maintain engagement with the counting task (away from internal sensations). Counting aloud allowed researchers to verify compliance with the distracting strategy.

### 2.6. Statistical Analysis

Analyses were conducted using IBM SPSS 27. Descriptive statistics were examined to assess normality and identify outliers. Independent samples *t*-tests were used to evaluate group differences in manipulation check variables and outcome measures. Primary analyses focused on between-condition differences in state mindful pain reappraisal, with secondary, exploratory analyses examining pain severity, pain tolerance, affect, and perceived exertion. Potential covariates including gender, exercise order, baseline trait variables, prior mindfulness experience, reported ability to use the assigned strategy, and amount of strategy practice were examined using analysis of covariance (ANCOVA). Only covariates demonstrating significant associations with outcome variables were retained in final models.

## 3. Results

### 3.1. Descriptive Statistics

All variables were normally distributed and no outliers were identified. Baseline trait variables did not differ between conditions, supporting comparability of groups prior to attentional manipulation (Table 2) The number of form corrections required during each exercise was recorded as an index of task compliance. During the wall-sit, the number of form corrections did not differ between conditions (*p* = 0.30), with participants in the mindfulness condition requiring an average of 0.32 corrections (*SD* = 0.72) and those in the backward-counting condition requiring 0.54 corrections (*SD* = 0.78). During the plank, participants in the backward-counting condition (*M* = 1.29, *SD* = 1.40) required significantly more form corrections (*t* = 2.9, *p* = 0.006) than those in the mindfulness condition (*M* = 0.39, *SD* = 0.57). This difference may reflect greater attentional monitoring of body position in the mindfulness condition and could have influenced task performance or pain-related outcomes. This pattern would suggest that mindful attention may have supported greater internal monitoring of task execution during the plank, though this finding was exploratory and not a primary outcome.

Between sessions, participants were asked to practice their assigned attentional strategy. Participants in the mindfulness condition reported significantly more practice (*M* = 2.46, *SD* = 0.79) than those in the backward-counting condition (*M* = 2.00, *SD* = 0.93, *p* = 0.03) This difference indicates greater engagement with the mindfulness practice materials between sessions. Following each exercise, participants rated their ability to use the assigned strategy. During the wall-sit, self-reported ability did not differ between the mindfulness (*M* = 1.79, *SD* = 0.96) and backward-counting conditions (*M* = 1.50, *SD* = 0.89). During the plank, participants in the mindfulness condition (*M* = 1.93, *SD* = 1.02) reported significantly greater ability to use their strategy than those in the backward-counting condition (*M* = 1.33, *SD* = 0.76, *p* = 0.01). Despite these differences in perceived strategy engagement, manipulation check results confirmed that attentional focus was successfully differentiated between conditions. At the same time, the greater reported practice and perceived ability to use the mindfulness strategy suggest potentially higher engagement with the assigned task, which should be considered when interpreting these findings.

### 3.2. Manipulation Checks

Independent *t*-tests were run on state mindfulness and attentional focus scores to evaluate the manipulations in both conditions. There were no group differences (*p* > 0.05) at baseline. Following attentional manipulation, attentional focus was significantly (*p* < 0.001) more internally focused and state mindfulness was significantly (*p* < 0.001) higher in the mindfulness condition than in the backward-counting condition (Table 2). These results show that the acute mindfulness training and mindful cues were successful in inducing an internal state of attention. This also provides evidence that counting backward was a successful strategy in distracting attention away from the body. The effect sizes of both manipulation checks were large (*Cohen’s d* > 0.80). These results provide evidence that the condition protocols were successful in manipulating attention which enhances the internal validity of the experiment. The lack of differences at baseline support baseline as a control (no manipulation) condition.

### 3.3. Hypothesis Testing

Independent *t*-tests showed there were no significant (*p* > 0.05) differences in core affect, pain severity, pain tolerance, pain reappraisal, or RPE at baseline. This is important to note as the groups were at the same starting point prior to the manipulation. To explore possible covariate effects, ANCOVA was conducted testing trait variables of mindful pain reappraisal, trait mindfulness as well as gender, exercise order, mindfulness/meditation experience, reported ability to use the assigned strategy during the experimental session, and the amount of practice of the given strategy between sessions one and two were explored as potential confounding variables. Of these covariates, only the reported ability to use the assigned strategy during the experimental session (*p* < 0.001) and the amount of reported practice of their given strategy between sessions (*p* = 0.01) were significantly different between groups. However, despite significant group differences, these covariates were not significant (*p* > 0.05) in the ANCOVA model. Only significant covariates for each outcome were included in the final models. Of all the covariates, only trait mindful pain reappraisal was a significant (*p* < 0.001) covariate in the models testing mindful pain reappraisal for plank and wall sit and exercise order was a significant (*p* = 0.047) for plank pain. ANCOVA results are in Table 3. Following experimental manipulation there was a significant (*p* = 0.04, η_p_^2^ = 0.08) difference in pain severity while doing a plank between the mindfulness (*M* = 2.59, *SD* = 1.64) and backward-counting (*M* = 3.56, *SD* = 1.84) conditions. There was a significant difference between mindful pain reappraisal in the wall-sit (*p* = 0.01, η_p_^2^ = 0.16) between the mindfulness (*M* = 4.11, *SD* = 1.36) and backward counting (*M* = 3.19, *SD* = 1.26) conditions. There was also a significant difference between mindful pain reappraisal in the plank (*p* = 0.02, η_p_^2^ = 0.14) between the mindfulness (*M* = 4.04, *SD* = 1.27) and backward-counting (*M* = 3.19, *SD* = 1.26) conditions. These results provide supporting evidence that an acute bout of mindfulness training and mindful cueing during exercise was associated with differences in pain reappraisal and lower pain severity during the plank task.

## 4. Discussion

The purpose of this study was to examine how different modes of attentional engagement shape the cognitive appraisal of exercise-induced pain. By contrasting a mindful associative strategy with an active, cognitively demanding dissociative strategy during isometric exercise, this study aimed to clarify how attention influences pain experiences during physical effort, rather than whether pain is simply avoided or reduced. Manipulation checks confirmed robust differentiation of attentional focus and state mindfulness between conditions, supporting the internal validity of the experimental design.

Consistent with hypotheses, participants using mindful attention demonstrated greater mindful reappraisal of pain during both the plank and wall-sit exercises compared to those using backward counting. This finding suggests that mindfulness is associated with a shift in how exercise-induced pain is cognitively interpreted, supporting theoretical models that emphasize appraisal as a key mechanism of mindfulness-based analgesia (Garland et al., 2015; Garland et al., 2022). Importantly, this effect emerged following a brief, acute mindfulness exposure, indicating that pain appraisal is a malleable process even within short-term exercise contexts. It is also worth noting that participants in the mindfulness condition reported greater between-session practice and higher perceived ability to implement the strategy, which may reflect increased engagement and could have contributed to the observed differences in pain reappraisal.

Pain severity during the plank was significantly lower in the mindfulness condition, whereas no difference was observed during the wall-sit. This task-specific effect may reflect differences in familiarity, postural demand, or interoceptive salience between exercises. Planks may afford greater opportunity for attentional monitoring and reinterpretation of discomfort, whereas wall-sits may elicit a more uniformly aversive or novel pain experience that constrains the influence of attentional strategy on perceived intensity. This effect may also be interpreted in light of the lower number of form corrections observed in the mindfulness condition during the plank, which may reflect enhanced attentional monitoring of body position and could have contributed to more stable task execution and, in turn, differences in pain perception.

Notably, no between-condition differences were observed for pain tolerance or core affect. These null findings are theoretically informative rather than contradictory, albeit somewhat exploratory. Pain tolerance in isometric tasks reflects a complex interaction of muscular endurance, motivation, and biomechanical constraints, and may be less sensitive to acute attentional manipulations than cognitive appraisal processes. Similarly, prior research suggests that both mindfulness and distraction can support comparable affective experiences during exercise (Cox et al., 2020; Jones et al., 2014), consistent with the present results.

This pattern suggests that attentional strategies may differentially influence the subjective experience of exercise without necessarily altering behavioral endurance. In applied contexts, this distinction may be particularly relevant for individuals who perceive exercise as aversive or difficult to tolerate. Strategies that modify how discomfort is interpreted, such as mindful attention, may support greater engagement by reducing the perceived threat or unpleasantness of bodily sensations. In contrast, distraction-based strategies may be more useful in situations where temporarily shifting attention away from discomfort is desirable, such as during unfamiliar or highly demanding tasks. Different attentional strategies may serve different functional roles depending on the context and goals of the individual.

Together, these findings suggest that mindfulness may not operate primarily by disengaging attention from bodily sensations during exercise. Rather, mindful attention may influence how pain is evaluated while maintaining engagement with interoceptive cues. Whereas distraction reduces awareness of discomfort by shifting attention away from the body, mindfulness allows pain sensations to be experienced without amplification through negative appraisal. This distinction is central to understanding how attentional strategies shape exercise experiences and align with contemporary models of pain as a perceptual and cognitive phenomenon rather than a purely sensory one. This interpretation is also consistent with predictive accounts of perception, which suggest that attention shapes how sensory input is interpreted rather than simply increasing or decreasing awareness. In this context, mindful attention may alter how interoceptive signals are evaluated and weighed without removing attention from them, though the specific mechanisms underlying this process were not directly examined in this study and warrant further investigation.

The dissociation between perceived pain and behavioral outcomes further supports the distinction between perceptual experience and task performance. Pain ratings reflect a subjective evaluation of bodily state, whereas endurance in isometric tasks is constrained by physiological and biomechanical limits that may not shift in parallel with perception. This suggests that attentional strategies can modify how pain is experienced without necessarily altering the physical thresholds that determine task termination. From this perspective, pain may function as a modulatory signal influencing subjective experience, rather than a direct determinant of behavior, highlighting the importance of distinguishing between perceptual and performance-based outcomes in exercise contexts.

The present findings also provide novel support for the use of the MRPS in exercise contexts. Trait mindful pain reappraisal was associated with state reappraisal during exercise, and state reappraisal differed systematically by attentional strategy. These results position mindful pain reappraisal as a promising mechanistic construct for understanding how attention shapes pain experiences during physical activity. Given that the MRPS was designed to capture appraisal processes rather than general mindfulness or coping, its sensitivity to acute attentional manipulation in this study supports its utility for future research in exercise and movement settings.

Several limitations warrant consideration. The absence of a no-strategy control group limits conclusions and comparisons between attentional strategies rather than absolute effects of attention. Additionally, while mindfulness and backward counting were selected to impose comparable cognitive demands, subjective and objective indices of cognitive load were not directly assessed. It is therefore possible that the two strategies differed not only in attentional orientation (internal vs. external), but also in the type or magnitude of cognitive load imposed. Variations in cognitive load may influence pain perception by altering the availability of attentional resources for processing nociceptive input, which should be considered when interpreting the observed differences between conditions. The laboratory context and structured cueing may also limit generalizability to naturalistic exercise environments. There were multiple outcomes tested, which could increase the chance of error. The secondary outcomes assessed could be considered more exploratory and results need to be replicated. Finally, the proposed mechanisms are inferred primarily from self-report measures, including a state-adapted post-task assessment, which limits the strength of mechanistic conclusions that can be drawn. Future studies incorporating extended training, objective cognitive load measures, and ecologically valid exercise settings may further clarify attentional mechanisms underlying pain appraisal during physical activity.

## 5. Conclusions

This study demonstrates that mindful attention is associated with differences in how exercise-induced pain is cognitively appraised, without corresponding changes in affective experience or behavioral endurance. Even following a brief, acute manipulation, participants using mindful attention showed greater capacity to reinterpret pain interoceptive cues in a non-threatening manner, as indexed by mindful pain reappraisal. Reduced pain severity during the plank task further suggests that shifts in appraisal may influence perceived pain intensity under certain task conditions. Together, these findings highlight cognitive pain appraisal as a key mechanism through which attentional engagement shapes exercise-induced pain experiences. By focusing on how attention is engaged rather than whether pain avoided, this work provides a framework for understanding how mindfulness may support sustained engagement with physically demanding activity without disengaging from bodily signals essential for performance and safety.

## Figures and Tables

**Table 1:** Descriptive statistics and demographics.

	Associative (*n* = 28)	Dissociative (*n* = 27)
Level of Mindfulness Practice (%)		
Beginner	17.9	15.4
Beginner-Intermediate	14.3	23.1
Intermediate-Advanced	7.1	0.0
NA (do not practice)	60.7	61.5
Bodily Pain (%)		
None	17.9	18.5
Mild	64.3	63.0
Moderate	17.9	18.5
* Type of Exercise Performed Weekly (%)		
Cardio	92.9	96.3
Muscular Strength	71.4	70.4
Muscular Endurance	39.3	48.1
Isometrics	14.3	14.8

* Participants were instructed to list every type of exercise they perform regularly, which is why values are over 100%.

**Table 2:** Trait variables and manipulation checks.

Baseline Variables:	Mindfulness		Backward Counting		*t*	*p*	*Cohen’s d*
	M	SD	M	SD			
Trait Mindfulness	3.44	0.45	3.22	0.53	1.70	0.05	0.49
Trait Mindful Pain Reappraisal	3.72	1.20	3.58	0.98	0.49	0.31	0.13
Manipulation Checks PL:							
Attentional Focus	18.75	15.75	65.22	21.13	−9.27	<0.001	−2.50
State Mindfulness	2.74	0.57	1.91	0.64	5.02	<0.001	1.35
Manipulation Checks WS:							
Attentional Focus	17.82	15.75	67.96	17.43	−10.9	<0.001	−3.03
State Mindfulness	2.87	0.52	1.96	0.62	5.89	<0.001	1.59
WS = Wall-sit, PL = Plank							

**Table 3:** ANCOVA results comparing mindfulness and backward-counting conditions.

	Mindfulness		Backward Counting		*F*	*p*	Eta Squared
	M	SD	M	SD			
Wall Sit:							
Core Affect	0.65	2.00	0.79	2.04	0.07	0.79	0.001
	95%CI (−0.12–1.41)		95%CI (0.01–1.57)				
Pain Severity	3.19	1.91	3.61	1.94	0.66	0.42	0.01
	95%CI (2.46–3.92)		95%CI (2.87–4.35)				
Change in Pain Tolerance *	3.96	24.49	9.26	37.40	0.39	0.54	0.01
	95%CI (−7.97–15.90)		95%CI (−2.90–21.41)				
RPE	14.32	2.37	14.59	2.21	0.20	0.66	0.004
	95%CI (13.45–15.19)		95%CI (13.71–15.48)				
Mindful Pain Reappraisal	4.11	1.36	3.19	1.26	9.56	0.01	0.16
	95%CI (3.69–4.41)		95%CI (2.89–3.62)				
Plank:							
Core Affect	0.82	1.83	0.70	2.13	0.05	0.83	0.001
	95%CI (−0.95–1.19)		95%CI (−1.19–0.95)				
Pain Severity	2.59	1.64	3.56	1.84	4.28	0.04	0.08
	95%CI (1.91–3.25)		95%CI (2.89–4.25)				
Change in Pain Tolerance *	0.07	19.61	6.41	24.79	1.11	0.42	0.02
	95%CI (−8.38–8.52)		95%CI (−2.2–15.01)				
RPE	14.00	2.14	14.43	1.83	0.63	0.43	0.01
	95%CI (13.24–14.76)		95%CI (13.66–15.20)				
Mindful Pain Reappraisal	4.04	1.27	3.19	1.24	8.36	0.01	0.14
	95%CI (3.63–4.34)		95%CI (2.89–3.61)				

Note. * This was the average change in pain tolerance, measured in seconds, from baseline to experimental session. The following covariates were included: The plank pain model included exercise order. The Plank and Wall Sit Mindful Pain Reappraisal models included Trait Mindful Pain Reappraisal. The 95% confidence intervals are shown below each mean.

## Data Availability

The data presented in this study are available on request from the corresponding author.
